# Effects of experimental watering but not warming on herbivory vary across a gradient of precipitation

**DOI:** 10.1002/ece3.7197

**Published:** 2021-01-27

**Authors:** Adam Pepi, Richard Karban

**Affiliations:** ^1^ Graduate Group in Ecology University of California Davis CA USA; ^2^ Department of Entomology and Nematology University of California Davis CA USA

**Keywords:** Bodega Marine Reserve, climate gradient, Humboldt Bay National Wildlife Refuge, open‐top chamber, precipitation

## Abstract

Climate change can affect biotic interactions, and the impacts of climate on biotic interactions may vary across climate gradients. Climate affects biotic interactions through multiple drivers, although few studies have investigated multiple climate drivers in experiments. We examined the effects of experimental watering, warming, and predator access on leaf water content and herbivory rates of woolly bear caterpillars (*Arctia virginalis*) on a native perennial plant, pacific silverweed (*Argentina anserina ssp. pacifica*), at two sites across a gradient of precipitation in coastal California. Based on theory, we predicted that watering should increase herbivory at the drier end of the gradient, predation should decrease herbivory, and watering and warming should have positive interacting effects on herbivory. Consistent with our predictions, we found that watering only increased herbivory under drier conditions. However, watering increased leaf water content at both wetter and drier sites. Warming increased herbivory irrespective of local climate and did not interact with watering. Predation did not affect herbivory rates. Given predictions that the study locales will become warmer and drier with climate change, our results suggest that the effects of future warming and drying on herbivory may counteract each other in drier regions of the range of *Argentina anserina*. Our findings suggest a useful role for range‐limit theory and the stress‐gradient hypothesis in predicting climate change effects on herbivory across stress gradients. Specifically, if climate change decreases stress, herbivory may increase, and vice versa for increasing stress. In addition, our work supports previous suggestions that multiple climate drivers are likely to have dampening effects on biotic interactions due to effects in different directions, though this is context‐dependent.

## Graphical abstract

Multiple interacting climate drivers are expected to affect ecological interactions and to vary across climate gradients. In this study, we manipulate temperature and moisture a two sites across a gradient of precipitation and find that warming increases herbivory at both sites, but watering only increases herbivory at the drier site. These findings are consistent with predictions drawn from range‐limit theory and the stress‐gradient hypothesis.

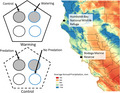

## 1 INTRODUCTION

Climate change is expected to significantly affect species interactions and geographic ranges (Alexander et al., 2016; Parmesan, 2006; Walther et al., 2002). These effects may be due to direct effects of changes in temperature or precipitation on organisms, or due to changes in species interactions (Tylianakis et al., 2008). The effects of climate change on species interactions are difficult to predict, as they are expected to vary geographically (Bertness & Callaway, 1994; Louthan et al., 2015; Silliman & He, 2018). Range‐limit theory (e.g., the species interaction‐abiotic stress hypothesis [SIASH]; Louthan et al., 2015) and the stress‐gradient hypothesis (SGH) both predict population‐level effects of biotic interactions to trade off with effects of abiotic stress (Bertness & Callaway, 1994; Silliman & He, 2018). However, if climate change has a variable influence on per capita effects of biotic interactions across stress gradients, this may complicate or counteract some of the predictions of SIASH or SGH. Here, we define stress as environmental conditions negatively impacting an organism's performance at the individual or population level. Herbivory is an important interaction affecting the distribution and abundance of plants (Maron & Crone, 2006), but studies examining whether increasing stress causes increasing or decreasing herbivory rates have had conflicting results (Chase et al., 2000; Cyr & Pace, 1993).

Drought stress is an important driver of plant distributions (e.g., Harrison et al., 1971) and is projected to increase (or has already increased) in some regions with climate change, in particular in California where this study was conducted (Bedsworth et al., 2018; Li et al., 2014). Drought or water stress has been hypothesized to affect insect herbivory rates on plants: Early research suggested that herbivores perform better on drought‐stressed plants (Mattson & Haack, 1987), while later work suggested that drought stress had neutral or negative effects on herbivore performance (Huberty et al., 2004). Most research has examined herbivore performance and not herbivory rates in response to drought, though it seems likely that greater herbivore performance would increase herbivory rates. However, a review examining specifically the effects of drought stress on damage rates to trees by insect herbivores found differing effects by herbivore guilds, with leaf‐chewing herbivores causing greater damage and wood‐boring insects causing less damage with greater water stress (Jactel et al., 2012).

The metabolic rate and feeding rate of herbivorous insects are temperature‐dependent (Bale et al., 2002), and therefore, climate warming has been predicted to increase rates of damage caused by insect herbivory (Wolf et al., 2008). This prediction would accord with the classical ecological expectation that herbivory rates and interaction strength should be stronger in warmer climates, that is, the tropics (Dobzhansky, 1950). However, meta‐analyses have not consistently supported the hypothesis that herbivory is higher in the tropics (Moles et al., 2011). Paleoecological research has suggested that herbivory rates in fossil plants have been higher during warmer periods over geologic time (Currano et al., 2008). Warming effects on metabolic rates in ectotherms are also expected to affect predator–prey interactions (Culler et al., 2015; Dell et al., 2014; Pepi et al., 2018), with some studies finding increased survival of prey (Culler et al., 2015) and others finding decreased survival of prey (Pepi et al., 2018).

Research on the effects of climate change on species interactions has typically focused on single climate drivers; however, there is the potential for interactive effects that would be overlooked in such studies (Scherber et al., 2013). Warming and drought together might interact to increase insect herbivory to even greater levels than either factor would cause in isolation (Jamieson et al., 2012). However, one meta‐analysis found that the interactive effects of multiple climate drivers generally exhibited dampening effects (Leuzinger et al., 2011). In the case of drought and warming on insect herbivory, it might be expected that each would drive effects in opposite directions. If drought has negative effects on insect herbivore performance, as has been found for some insect groups (Huberty et al., 2004; Scriber, 1979), then drought may compensate for the positive effects of warming on insect herbivory rates.

In the present study, we examined the effects of experimental warming and water addition on herbivory rates on a native perennial plant, pacific silverweed (Rosaceae: *Argentina anserina ssp. pacifica*), which occurs along the Pacific coast from California to Alaska. We conducted an experiment at two locations across a gradient of precipitation on the California coast, to test whether the effects of experimentally altered abiotic stress (i.e., water availability) on herbivory varied with baseline average seasonal abiotic stress. We also manipulated predator access to test whether warming affected predation rate and thus herbivory.

We hypothesized that:

1. Increased water availability increases leaf water content and herbivory but only under dry conditions when herbivores are water‐limited.
2. Warming increases herbivory rates due to increased metabolic rates.
3. Predator access will reduce herbivory and counteract increased metabolic rates of herbivores.
4. The effects of watering and warming interact to increase herbivory.

## 2 MATERIAL AND METHODS

To test the effects of water availability and temperature on herbivory rates on Pacific silverweed across a gradient of temperature and precipitation, experimental treatments simulating climate warming and increased precipitation were implemented at two sites. Experiments were set up in California in wet coastal prairie habitat, at Bodega Marine Reserve in the south (38°19′07″N, 123°04′15″W) on 3–5 July 2019 and Humboldt Bay National Wildlife Refuge in the north (40°41′13″N, 124°12′16″W) on 11–13 July 2019. Both sites have Mediterranean climates, with dry summers and cool wet winters. The northern site has an average annual precipitation about 30% greater than the southern site (1,114 mm vs. 859 mm) and June–September precipitation about 3.3× that of the southern site (51.0 mm vs. 15.6 mm) and an average annual temperature 0.5°C lower than the southern one (12.1°C vs. 12.6°C; all climate data are interpolated 1981–2010 normals from PRISM, Oregon State University). Both sites are wetland locations, with seasonally high water tables, commonly flooding during the winter in wetter years.

Pacific silverweed (*Argentina anserina ssp. pacifica*) is a perennial herbaceous plant that grows on the Pacific coast from southern California to Alaska, on well‐watered coastal bluffs, wetlands, stream banks, or other moist open areas (Pojar & MacKinnon, 1994). Pacific silverweed is a subspecies of common silverweed (*Argentina anserina*) which has a Holarctic distribution (Pojar & MacKinnon, 1994). It senesces in fall or winter, persisting overwinter as rhizomes. It emerges in late winter and spring, and spreads via stolons in the spring and early summer. It produces flowers and fruits in the summer. In California, it experiences seasonal drought in the summer and fall, more intensely at the southern than northern site.

**FIGURE 1:**
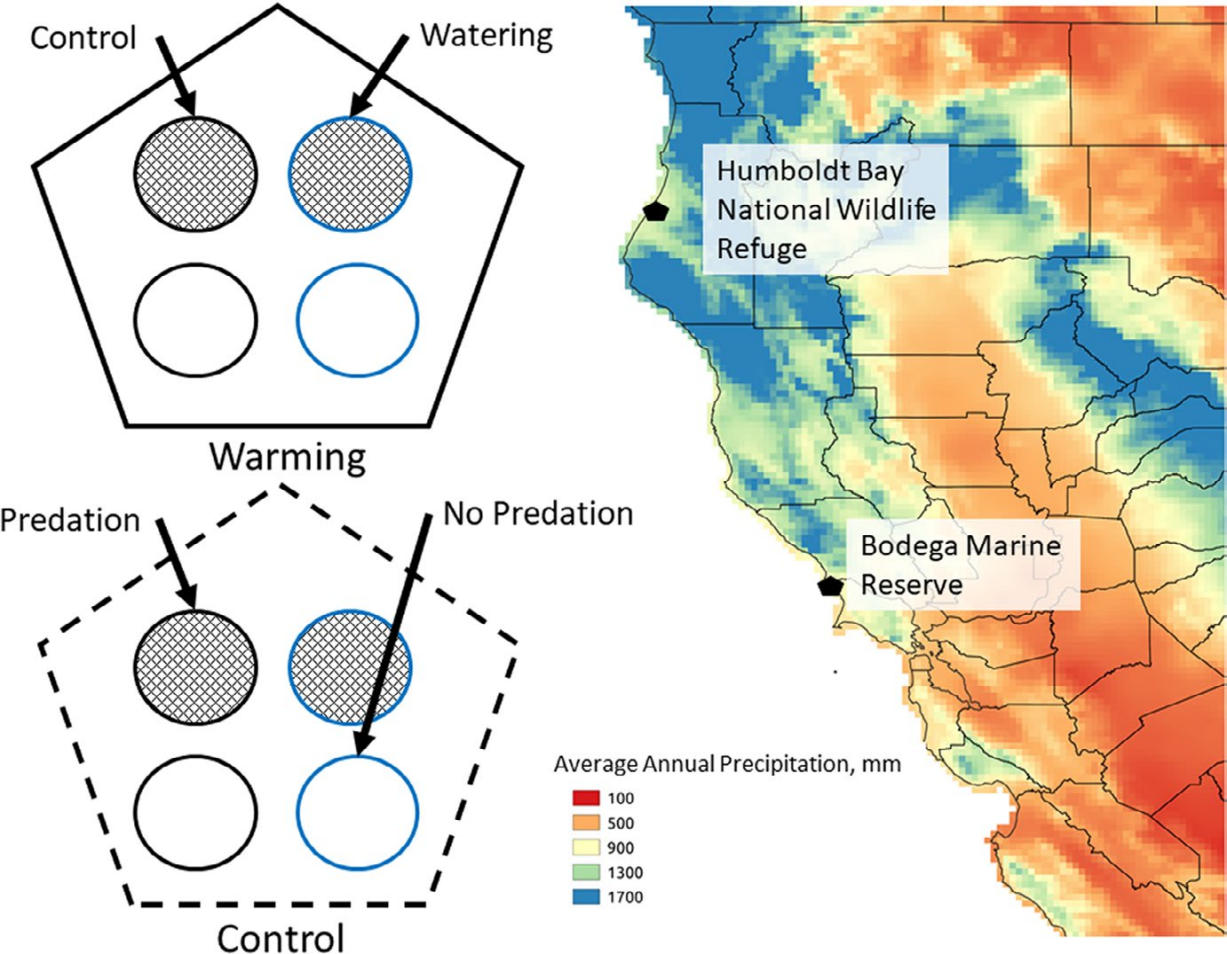
Schematic of the experimental design, and the study locations overlaid on a map of average annual precipitation. The schematic is representative of a single block: Half of the enclosures in the block are within the pentagonal OTC warming treatment, and half are outside. Half of the enclosures are watered, and half are control. Half have spun polyester bags (no predation), and half have window screening bags (predation). The map has outlines of California counties, and average annual precipitation from 1980 to 2010, interpolated by PRISM (Oregon State University)

Enclosures were constructed to control levels of herbivory around ca. 5–15 naturally occurring plants of roughly similar total biomass (visually estimated) in wet coastal prairie habitat. Enclosures were 45 cm diameter circles made of 20 or 25 cm wide aluminum flashing (Gibraltar Building Products) set ca. 10 cm into the ground. Sixty‐four enclosures were constructed, 32 at each site, arranged into 4 blocks at each site with 8 enclosures per block. Watering, warming, and predation treatments were applied in a split‐plot design, resulting in 8 treatment combinations, with one of each combination in each block (see Figure 1).

Five 2nd‐instar *Arctia virginalis* caterpillars were placed into each enclosure, and sleeves of either spun polyester (lightweight floating row cover) or window screening (1.13 mm × 1.30 mm openings) were secured to the rims of enclosures (using Gorilla duct tape; Gorilla Glue). *Arctia virginalis* is a species of tiger moth that is abundant at both sites and commonly feeds on Pacific silverweed, in addition to many other host plants (English‐Loeb et al., 1993). *A. virginalis* is univoltine, hatching from eggs in mid‐summer, overwintering as caterpillars, and pupating and emerging as adults to mate in the following spring or summer. *A. virginalis* is preyed upon by the ant *Formica lasioides* (Pepi et al., 2018)*,* and the spun polyester treatment excluded ants, whereas the window screening allowed ant access. Both enclosures contained *A. virginalis*. Enclosures also contained other herbivores, including an unidentified leaf beetle. Beetles were trapped in spun polyester enclosures but were able to move in and out of window screening.

For watering treatments, an automatic irrigation system was constructed at each site, connected to hose faucet timers (Orbit) with pressure regulators (25 PSI; Rain Bird). One main polyester tube (half inch diameter; Rain Bird) supplied each experimental block, attached to 6.35‐mm tubes extending into watered enclosures, with adjustable drip ends (Rain Bird). Irrigation timers were set to release ~3.8 L in each enclosure every 5 days at 08:00 PST for 1 hr, for the duration of the experiment, which was during the annual seasonal drought in the summer and fall at study sites. We used a watering treatment to create wetter summer soil conditions that were historically more common in coastal California. Dryer conditions are expected to continue to be common in California in the future (Li et al., 2014).

For passive warming treatments, pentagonal open‐top chambers (OTCs) were constructed based on designs from the International Tundra Experiment (Marion et al., 1997). OTCs were 2 m wide × 0.6 m high, constructed from clear polycarbonate roofing panels (SUNTUF; Palram Americas) secured with zip ties. To measure the effectiveness of warming treatments, temperature loggers (iButtons; Maxim Integrated Products, Inc.) were placed inside enclosures, attached to metal flashing, in one warmed and one control enclosure per block. Due to poor measurements from this arrangement, loggers were also placed inside shields (PVC tubing, 6 cm diam × 15 cm length) at the center of the warmed and control section of each block after 19 October 2019 at Humboldt Bay National Wildlife Refuge and after 9 November 2019 at Bodega Marine Reserve. Loggers in shields recorded 0.8°C warming due to OTCs at Humboldt and 0.4°C warming at Bodega from October/November to December. Overall, we expect that warming due to OTCs ranged from ~0.4 to 1.5°C over the season (see Appendix S1).

To measure herbivory rates on plants in enclosures, recently frost‐killed leaves were collected haphazardly from enclosures on 6 December 2019 at Humboldt Bay National Wildlife Refuge and 7 December 2019 at Bodega Marine Reserve. We expect that this introduced little bias because leaves were killed only ~1 week prior to collection by an unusual cold event. Identifying individual plants was not feasible due to the clonal nature of their spread. Herbivory rates were measured as the proportion of damaged leaflets out of 10 leaves, or as many as present if fewer than 10 (total *N* = 573; average number of leaves per enclosure = 8.9). Herbivory was relatively evenly spread across leaflets, and therefore, we do not expect that measuring the proportion of damaged leaflets instead of area consumed biased our estimates. We were unable to assess treatment effects on *Arctia virginalis* caterpillars due to very low survival (3 caterpillars out of 325). The proportion of leaflets with damage was analyzed in beta‐binomial generalized linear mixed effect models to account for overdispersion with a nested random effect of enclosure and block to account for spatial nonindependence of herbivory rates. A set of hypothesized models was generated including fixed effects of watering, warming, and predation treatments, site, and interactions (Table 1). Interactions between watering, warming, and site were included in multiple models which were compared using the Akaike information criterion (AIC). We included models with predation treatment because it was included in the experimental design although that variable had poor predictive power. We also present more parsimonious models without the predation treatment for our ultimate results.

**TABLE 1:** Models of herbivory considered in model selection

Model structure	df	ΔAIC	AIC	AIC weight
**y ~ watering × site + warming + (1|enclosure/block)**	**8**	**0.00** *	**1,262.98** *	**0.51**
**y ~ watering × site + warming + predation + (1|enclosure/block)**	**9**	**1.72**	**1,264.69**	**0.22**
y ~ watering × site + warming × site + predation + (1|enclosure /block)	10	3.64	1,266.62	0.08
y ~ watering × site + warming × predation + (1|enclosure /block)	10	3.66	1,266.64	0.08
y ~ watering × warming + site + predation + (1 enclosure/block)	9	4.60	1,267.57	0.05
y ~ watering × warming × site + predation + (1|enclosure/block)	12	5.45	1,268.43	0.03
y ~ watering + site + warming × predation + (1|enclosure/block)	9	5.89	1,268.87	0.03

Note

All models use a beta‐binomial error distribution and include nested random effects of enclosure within block. Number of parameters, difference in the Akaike information criterion (AIC) relative to lowest AIC‐scored model (ΔAIC), AIC score, and AIC model weight are shown. The two models with ΔAIC < 2 are bolded.

* The best‐fit model is starred

Water content of leaf material pooled from 1 to 5 leaves collected on 19 October 2019 (Humboldt) and 9 November 2019 (Bodega) from each enclosure was measured by weighing fresh and dried leaf material. The proportion of moisture by weight was analyzed using beta‐binomial generalized linear mixed effect models, with enclosure and block as nested random effects. Site, watering, and warming treatments were included as fixed effects. In a separate model, an interaction between watering and site was tested in addition to the fixed effects.

All analyses were conducted in R version 3.5.1. Generalized mixed effect models were conducted using the package GLMMTMB (Brooks et al., 2017), back‐transformed model effects were calculated using EMMEANS (Lenth, 2019), and plots were generated using GGPLOT2 (Wickham, 2016). In general, we report all results from our analyses, whether below the *p* = .05 threshold or not, to avoid problems that arise from using *p* = .05 as a cutoff (Wasserstein & Lazar, 2016). Therefore, we present all our results and allow readers to assess the weight of the evidence.

## 3 RESULTS

**FIGURE 2:**
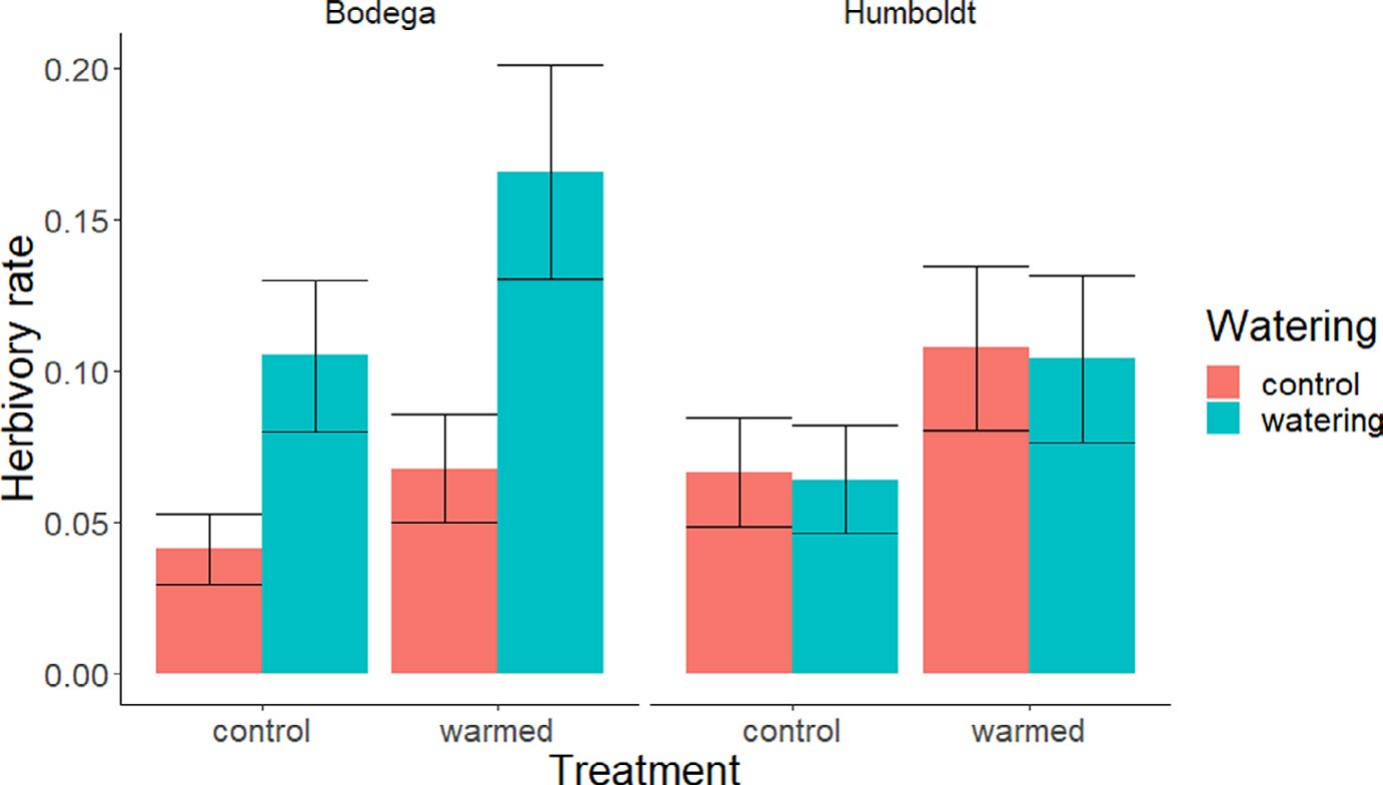
The model‐derived predicted mean herbivory rate ± 1 *SE* for warming and watering treatments by site. The watering treatment increased herbivory at the southern, drier site (Bodega Marine Reserve) but not at the northern, wetter site (Humboldt Bay National Wildlife Refuge). Warming increased herbivory at both sites. Predictions are derived from the conditional model only (fixed effects; generated using the package EMMEANS)

Plants that were watered and warmed experienced greater herbivory. The best model structure describing herbivory included watering, warming, site, and an interaction between site and watering treatments. A model of the same structure but including the predation treatment was within Δ AIC < 2 of the best fitting model (Table 1). In the best fit model (Figure 2), watering was estimated to increase herbivory by 55% overall (logit *β* = 1.004, *z* = 2.965, *p* = .003), and warming was estimated to increase herbivory overall by 62% (logit *β* = 0.525, *z* = 2.114, *p* = .037). Effects of the treatments depended upon the site. Mean herbivory rates were higher at Humboldt (60% higher in controls), but this difference may have been due to chance (logit *β*_Humboldt_ = 0.5066, *z* = 1.419, *p* = .1558). However, an interaction between watering treatments and site showed that watering increased herbivory at the drier Bodega by 150% but not at the wetter Humboldt (3% decrease; logit *β*_Humboldt _= −1.004, *z *= −2.085, *p* = .037).

Additional interactions that were tested had limited statistical support. The interaction between warming treatments and site estimated a 22% greater effect of warming at Humboldt than Bodega but was most likely due to chance (logit *β*_Humboldt_ = 0.136, *z* = 0.274, *p* = .784). There was a positive interaction between watering and warming, with herbivory estimated to be 60% greater with both watering and warming than just watering and 54% greater than just warming, although there was a significant probability that this effect was due to chance (logit *β* = 0.598, *z* = 1.174, *p* = .240). Due to overdispersion of the response variable, the power of the model to detect effects of interactions was likely limited. A power analysis (package SIMR; Green & Macleod, 2016) of a version of the model refit using LME4 (Bates et al., 2014) with binomial error structure found only 45% power to detect an interaction between watering and warming at the estimated effect size. To reach a standard of 80% power to detect an effect, a very large effect size (logit *β* ≈ 1.375) would be necessary given the experimental design and overdispersed response variable.

These effects of climate on rates of herbivory were not affected by predation or lack thereof; predation treatments had little effect in any model. The no predator access treatment had 11% lower herbivory, but that effect was most likely due to chance (logit *β *= −0.132, *z* = −0.535, *p* = .592). The interaction between warming and predation also had little effect and was in the opposite of the expected direction; the effect of warming was 18% less in the no predation treatment than the predation treatment, and this difference was most likely due to chance (logit *β* = −0.111, *z* = −0.225, *p* = .822).

**FIGURE 3:**
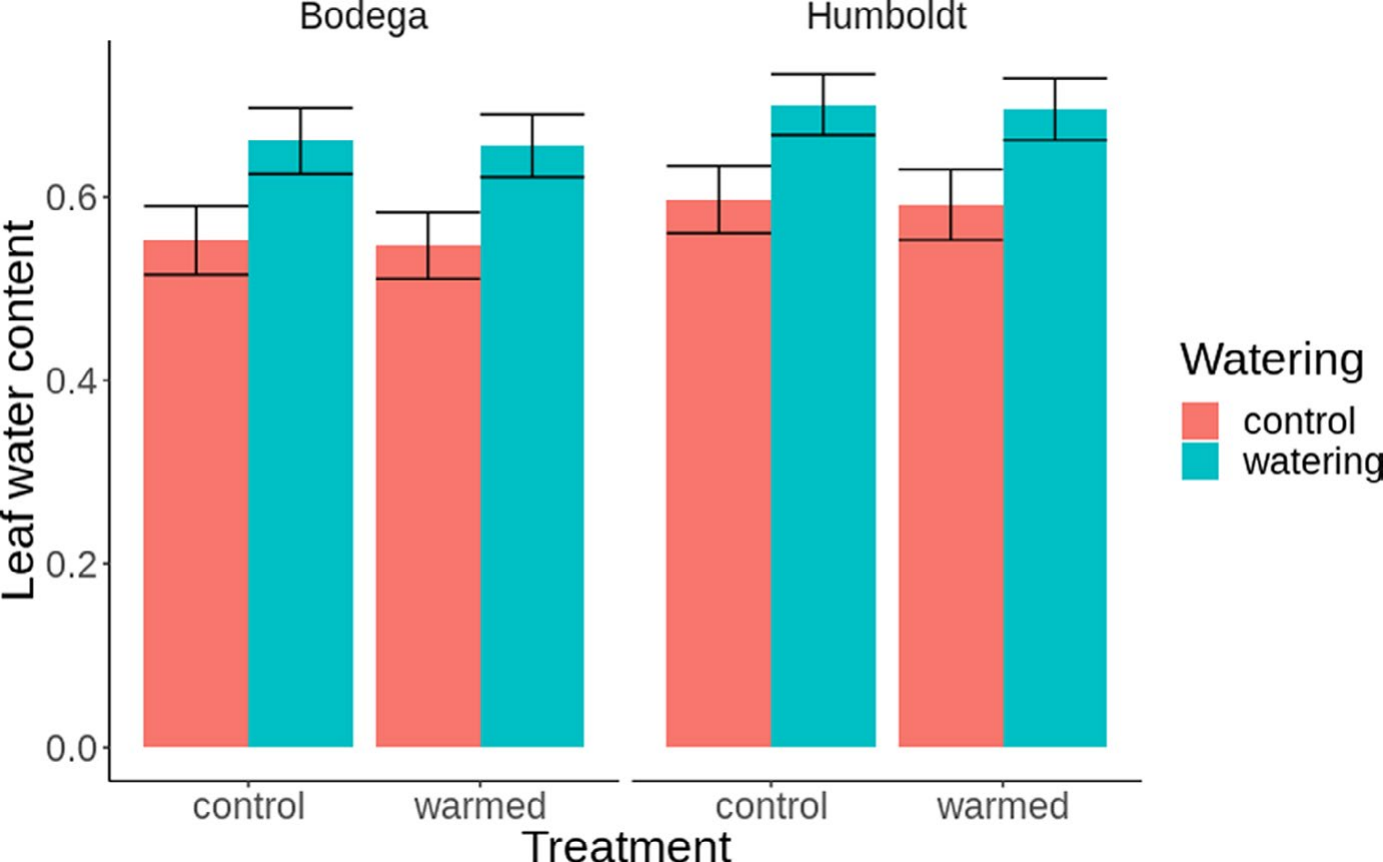
The model‐derived predicted mean leaf water content by proportion of weight ± 1 *SE* for warming and watering treatments by site. The watering treatment increased leaf water content, and the warming treatment had no effect. Predictions are derived from the conditional model only (fixed effects; generated using the package EMMEANS)

Watering treatments increased leaf water content by 19% (logit *β* = 0.457, *z* = 2.953, *p* = .003; Figure 3), but warming had no effect (logit *β* = −0.023, *z* = −0.150, *p* = .881). Leaf moisture content was 7% higher at Humboldt but may have been due to chance (logit *β*_Humboldt_ = 0.18199, *z* = 1.175, *p* = .240). The interaction between site and watering treatment estimated an additional small effect of watering at Humboldt of 11.4% but may have been due to chance (logit *β*_Humboldt_ = 0.31, *z* = 1.047, *p* = .295).

## 4 DISCUSSION

We found that herbivory was increased by water addition at the drier southern site, while watering had no effect at the wetter northern site. Warming increased herbivory at both sites; there was evidence suggestive of a positive interaction between watering and warming treatments, although our power to detect this interaction was limited. Predation treatments had no effect, suggesting that there was little predation occurring and that warming had no effect on predator–prey interactions. Placing ant baits at experimental sites found very few ants as well, supporting the absence of significant ant predation.

Watering also increased leaf water content, potentially providing a mechanism for increased herbivore performance and higher herbivory rates at the southern site (Scriber, 1979). Contrary to our predictions, there was no evidence of a corresponding negative interaction between site and watering treatment for leaf water content as there was for herbivory. However, there was a trend toward higher leaf water content at Humboldt; if there was a nonlinear, positive response of leaf water content on herbivore performance, this might explain the difference in watering treatments effects between Bodega and Humboldt. Leaf water content was measured at a single time point over a five‐month experiment; differences between sites might also have been greater earlier in the summer when greater evapotranspiration levels were occurring. During the summer, it is also possible that plants in watering treatments invested more in growth than in defense. Younger insects are more likely to be negatively affected by plant quality than older individuals which occur later in the season (Zalucki et al., 2002). A nonlinear decline of defense investment in response to water availability might also have resulted in the differences between the effects of the watering treatment between Humboldt and Bodega.

Our results suggest that consumer control (herbivory) may trade off with abiotic stress in this species due to reduced insect performance on plants in drier conditions. This finding accords with the predictions of SGH and SIASH, though through a different mechanism, since both SGH and SIASH assume that biotic interactions become limiting in favorable environmental conditions due to greater population growth of both consumers and resources (Bertness & Callaway, 1994; Louthan et al., 2015; Silliman & He, 2018). However, our study only examined a potential correlate of consumer control (herbivory), as we did not measure fitness or population growth. We also only conducted our study at two sites over a small portion of the range of Pacific silverweed which limits our ability to generalize across the range or precipitation gradients. We attempted to account for this by selecting sites that were as similar as possible, but it is still possible that differences between sites were due to factors other than a climate gradient. Lastly, we were not able to separate direct versus indirect effects of treatments on plants because we did not include a control without herbivores. Overall, our experimental design involved a compromise between external and internal validity (Naeem, 2001). Our design created external validity because it was a field experiment at two sites along a precipitation gradient, conducted over a 5‐month period, using experimental mesocosm‐scale enclosures. Our design created internal validity because it manipulates warming, watering, and predator access, allowing us to understand some of the mechanisms driving our results.

California's climate is expected to become warmer and drier in the future (Li et al., 2014). Our findings suggest that warming and drying are likely to have opposing effects on herbivory for Pacific silverweed, possibly negating any climate effects on herbivory rates for this species toward the southern range edge. We could find surprisingly few experiments that have simultaneously manipulated both temperature and water and measured effects on herbivory. The one study we were able to find that examined effects of both factors on herbivore performance found that both warming and drought had negative effects on herbivore performance and thus presumably on herbivory rates (Scherber et al., 2013); this result contrasts with the results of the present study in which effects of warming and drought were opposite. The findings of the present study accord with the general observation that studies examining multiple climate drivers often find that they exhibit dampening or opposing effects (Leuzinger et al., 2011), at least if the results of this study are projected into the future with predicted changes to California climate. However, we did find additive or potentially interactive effects of multiple climate drivers, in the sense that our results predict that warming coupled with increased precipitation would result in greatly increased herbivory on silverweed; these changes in climate are predicted in some parts of the range of the species (e.g., the Pacific Northwest [Mote & Salathé, 2010]).

Our findings also suggest that projected future climate changes may have more negative effects on Pacific silverweed at the wetter center of its range than at the drier southern edge, in terms of herbivory. We found that summer drought lessened herbivory but only in already relatively dry conditions and that warming uniformly increased herbivory. Therefore, in more northerly parts of the range of Pacific silverweed, drier conditions may not be sufficient to reduce herbivory, but warmer conditions may be enough to increase herbivory rates. These differences are due to geographical variation in species interactions that are consistent with a relatively simple hypothesis such as SIASH, but they result in complex and nonintuitive variation in responses of species interactions to warming across climate gradients.

In summary, this study provides an example of the complexity that is involved in ecological responses to climate change when species interactions and abiotic stress are considered across climate gradients. Even for single interactions, such as herbivory in the present case, different climate factors may drive changes in different directions (Leuzinger et al., 2011). Furthermore, the effects of different climate drivers on biotic interactions are likely to vary across environmental gradients with the potential to set novel range limits and novel species interactions.

## CONFLICT OF INTEREST

We declare no conflict of interest.

## AUTHOR CONTRIBUTION

**Adam Pepi:** Conceptualization (lead); Data curation (lead); Formal analysis (lead); Writing‐original draft (lead). **Richard Karban:** Conceptualization (supporting); Supervision (lead); Writing‐review & editing (supporting).

## Supporting information

Appendix S1

## Data Availability

The data and code associated with this study are archived at Knowledge Network for Biocomplexity (https://doi.org/10.5063/RJ4GV9).
