# Supplementary material for: Effects of experimental watering but not warming on herbivory vary across a gradient of precipitation
Source: Ecol Evol. 2021 Jan 27;11(5):2299–306. doi: 10.1002/ece3.7197 (PMC7920774; doi:10.1002/ece3.7197)
Supplement: Supplementary file 1 — Appendix S1 [file ECE3-11-2299-s001.docx]

**Supplement**

*Open Top Chamber (OTC) temperature effects*

To estimate the effectiveness of OTC treatments, we compared temperature logger readings from warming treatments and controls, using mixed effect models of hourly temperature readings with hour and block as random effects. We also conducted analyses to assess the effectiveness of treatments over time, including time scaled to months in an interaction with treatments, in mixed effect models with block as a random effect. Loggers attached to enclosures were present from July-Dec, and loggers in shields were present from Nov-Dec. In models without interactions (table S1), loggers in enclosures showed no net warming effect, although loggers in shields showed a net warming of 0.45±0.02°C at Bodega and 0.84±0.06°C at Humboldt from Nov-Dec. This showed that loggers placed in enclosures underestimated warming effects by ~0.44 –1.1°C. Analyses including temporal trends and interaction terms (table S2) showed that treatment effects tended to weaken over time, declining from ~0.2 – 0.6 °C per month. Based on effect sizes from shield loggers and temporal trends in treatment effects, we estimate the overall average warming effect to be between ~0.5 – 1.5 °C over the entire experiment.

| Site | Date | Logger position | β ± 1 SE | z | P |
| --- | --- | --- | --- | --- | --- |
| BMR | 6 July – 19 Sept | Enclosure | -0.41±0.04 | -10.40 | >0.0001 |
| HOS | 13 July – 6 Oct | Enclosure | 0.18±0.03 | 6.37 | >0.0001 |
| BMR | 9 Nov – 6 Dec | Shield | 0.45±0.02 | 18.50 | >0.0001 |
| BMR | 9 Nov – 6 Dec | Enclosure | -0.018±0.07 | -0.25 | 0.8 |
| HOS | 19 Oct – 7 Dec | Shield | 0.84±0.06 | 13.39 | >0.0001 |
| HOS | 19 Oct – 7 Dec | Enclosure | -0.184±0.062 | -2.94 | 0.0033 |
| BMR | 9 Nov – 6 Dec | Enclosure | -0.018±0.07 | -0.25 | 0.8 |

**Table S1.** Results of Gaussian linear mixed effect models of warming treatment effects as measured by temperature loggers, with spatial and temporal random effects.

| Site | Date | Logger position | Parameter | β ± 1 SE | z | P |
| --- | --- | --- | --- | --- | --- | --- |
| BMR | 6 July – 19 Sept | Enclosure | Treatment: Warmed | 0.19±0.22 | 0.86 | 0.387 |
|  |  |  | Time | -0.11±0.11 | -0.99 | 0.321 |
|  |  |  | Treatment*Time | -0.47±0.15 | -3.12 | 0.002 |
| HOS | 13 July – 6 Oct | Enclosure | Treatment: Warmed | 0.51±0.22 | 2.29 | 0.0222 |
|  |  |  | Time | -2.7±0.08 | -31.07 | >0.0001 |
|  |  |  | Treatment*Time | -0.24±0.13 | -1.78 | 0.075 |
| BMR | 9 Nov – 6 Dec | Shield | Treatment: Warmed | 0.64±0.29 | 2.19 | 0.028 |
|  |  |  | Time | -1.97±0.38 | -5.16 | >0.0001 |
|  |  |  | Treatment*Time | -0.42±0.54 | -0.77 | 0.44 |
| BMR | 9 Nov – 6 Dec | Enclosure | Treatment: Warmed | 0.29±0.21 | 1.36 | 0.175 |
|  |  |  | Time | -1.96±0.25 | -7.65 | >0.0001 |
|  |  |  | Treatment*Time | -0.60±0.36 | -1.66 | 0.096 |
| HOS | 19 Oct – 7 Dec | Shield | Treatment: Warmed | 1.34±0.29 | 1.13 | >0.0001 |
|  |  |  | Time | -1.03±0.22 | -4.65 | 0.135 |
|  |  |  | Treatment*Time | -0.62±0.31 | -1.98 | 0.048 |
| HOS | 19 Oct – 7 Dec | Enclosure | Treatment: Warmed | -0.035±0.37 | -0.094 | 0.925 |
|  |  |  | Time | -1.95±0.33 | -5.93 | >0.0001 |
|  |  |  | Treatment*Time | -0.19±0.40 | -0.46 | 0.644 |

**Table S2**. Results of Gaussian linear mixed effect models of warming treatment effects as measured by temperature loggers, with spatial random effects and a temporal trend (scaled to months).
